# Cystometric analysis of the transplanted bladder

**DOI:** 10.1590/S1677-5538.IBJU.2015.0117

**Published:** 2017

**Authors:** Jeová Nina Rocha

**Affiliations:** 1 Departamento de Urologia Hospital das Clínicas da FMRP-USP Ribeirão Preto, São Paulo, Brasil

**Keywords:** Transplantation, Urinary Bladder, Patients

## Abstract

**Objective:**

Cystometric evaluation of the bladder after autotransplant and isogeneic transplant in female rats.

**Material and Methods:**

Two groups were constituted: (A) bladder autotransplant with two subgroups: R1 – (control) and R2 – (bladder transplant); (B) isogeneic bladder transplant with three subgroups; T1 – (control); T2–T3, two subgroups observed for 30 and 60 days after transplant, respectively. All animals underwent cystometric evaluation. Afterwards, the bladders were removed for histological study.

**Results:**

The transplanted bladders did not show significant changes in filling/storage and emptying/micturition functions after 30 and 60 days of evolution. Upon macroscopical evaluation, there was good revascularization and the tissue was well preserved. Cystometry results: Did not show significant differences in the micturition pressure in subgroups T2-T3, but did between subgroups R1−R2, T1−T2, and T1−T3. Significant differences were verified in the micturition interval between T1−T3, T2−T3, but not between R1−R2, T1−T2. There was significant difference in the micturition duration between T1−T3 but not between R1−R2, T1−T2 and T2−T3. No fistula was noted on the suture site nor leakage of urine in the abdominal cavity or signs of necrosis or retraction were observed.

**Conclusions:**

Transplant of the bladder was shown to be a viable procedure. The results indicate that there was structural and functional regeneration of transplanted bladders, and these results indicate that it is possible that vascular endothelium growth and neurogenesis factors are involved and activated in the process of the preservation or survival of the transplanted organ.

## INTRODUCTION

A considerable number of patients requires augmentation or reconstructive cystoplasty to improve bladder urine storage and continence as well as to provide protection of the upper urinary tract. Diverse segments of the gastrointestinal tract, including gastric, small intestine and colon segments, have been employed for this aim (1-5). Alternative natural tissues taken from the uterus, peritoneum, lyophilized dura, pericardium and placenta have also been utilized for cystoplasty (6-12). Furthermore, synthetic materials such as Teflon, sylastic, polyvinyl sponges, poly-alpha amino or collagen/polyglactin membranes have also been tried (13-18). However, all these procedures are associated with many complications including metabolic and electrolyte disturbances, lithiasis, infections, neo-bladder spontaneous rupture or perforation and neoplasia (1-5). To avoid such complications, the use of living partial bladder transplantation has recently been proposed as an alternative procedure for bladder augmentation (5, 19, 20). Although this procedure was successful in increasing bladder capacity and long term survival of the transplants, it consisted of a two stage intervention and used only the cranial third of the donor bladder. It become of interest to determine whether a similar procedure using the whole supratrigonal bladder for transplantation and performed in a single operation was feasible. Furthermore, a more objective evaluation using a continuous cystometrogram of the transplanted bladder function was deemed necessary.

Consequently, the main objective of the present study was to examine the feasibility of supratrigonal bladder transplantation using autologous and syngeneic transplants. Once the feasibility of the procedure was documented a second objective was proposed to determine whether the transplanted bladders exhibit normal functions using cystometry.

## MATERIALS AND METHODS

Experiments were conducted using 45-50 days old Wistar and spontaneous hypertensive female rats (SHR). Wistar rats were employed for the partial bladder autotransplantation whereas SHR were used for the syngeneic transplant experiments. All the procedures followed recommendations of the Committee of Ethics in Animal Experimentation of the Ribeirão Preto Medical School–USP.

Supratrigonal bladder transplant experiments were initially performed to determine the feasibility of the surgical procedure. Female Wistar rats were anesthetized with tribromoethanol 2.5% (1mL/100g body weight), supplemented with additional doses as required. A low median laparotomy was performed to expose the urinary bladder; the supratrigonal segment of the bladder was excised avoiding any damage to the ureter entrances and placed immediately in a container with ice-cold NaCl solution (0.9g/100mL). After 30 min in this solution the excised supratrigonal bladder segment was sutured to the bladder base using continuous 7.0 polyglycolic acid sutures in both anterior and posterior faces leaving both ends of the sutures free. The free endings of both sutures were used to fix the omentum to the anterior and posterior faces of the reconstituted bladder. The abdominal wall incision was closed in two layers using 6.0 polypropylene monofilament. A PE-50 catheter was introduced into the reconstituted bladder through the urethra to allow urine drainage during the post-operative period. This catheter was fixed to the external urethral meatus with a 7.0 polyethylene suture and removed three days later under ether anesthesia. All animals received intrarectal acetaminophen (20mg/kg) after the surgical procedures. No immunosuppressive drugs were used. Once the feasibility of the surgical procedure was established syngeneic transplantation experiments were performed in SHR using a similar procedure.

After the surgical procedures, autotransplanted and syngeneic transplanted rats as well as age-paired control non-operated rats were kept under similar housing conditions (temperature 22±2ºC and 12:12h light/dark cycle) with free access to food and water.

### Continuous cystometrogram experiments

Thirty days after bladder autotransplantation or 30 and 60 days after syngeneic bladder transplantation both control and operated animals were weighed and anesthetized with urethane (1.2g/kg, subcutaneously). They were placed in a dorsal recumbent position and an incision was made on the anterior abdominal wall (for the operated rats, in the same site of the previous surgery) to expose the bladder (non-operated animals) or the reconstituted bladder (transplanted rats); one end of a PE-50 catheter, containing a small collar created by heating, was introduced into the bladder through a small incision in the bladder dome (including the omentum in the transplanted rats) and tied firmly to the bladder with a silk ligature. The other end of the catheter was linked via a T-connector to a pressure transducer (BLPR2, World Precision Instruments Inc, Sarasota, FL, USA) to record intravesical pressure and to a micro-infusion pump (Model 780.200, KD Scientific Inc. Holliston, MA, USA); warm saline was infused continuously at a rate of 0.08mL/min. Intravesical pressure data was recorded, stored and analyzed using the Windaq software (Dataq Instrument Inc. Akron OH, USA). The following cystometrogram parameters were measured: a) maximal micturition pressure (peak intravesical pressure developed during micturition contractions), b) micturition interval (time interval between the return of intravesical pressure [IVP] to baseline after a micturition contraction and the beginning of the next micturition contraction, i.e., time point at which the IVP showed a rapid increase), c) duration of micturition contractions, time lapsed from the beginning of the micturition contraction (see above) up to the return to baseline of the IVP.

#### Histology

After finishing the cystometrogram experiments the animals received an overdose of urethane i.p. The chest was opened to expose the heart and a cannula was inserted into the left ventricle and pushed up into the ascending aorta; an incision was also made in the right auricle to allow drainage and an intracardiac perfusion using cold saline (300mL) followed by buffered 4% paraformaldehyde (300mL) was performed. The whole bladder was then excised, immersed in the same fixative and kept at 4ºC until further processing. Bladders were embedded in paraffin and 10µm sections were mounted on microscopic slides and stained with hematoxylin-eosin or Masson’s trichrome stain. Slides were observed in an optical microscope and pictures were taken with a Microscopic Axiophot and Axiovision 4.8.1, Carl Zeiss Germany.

## Statistics

Body weight, maximal intravesical pressure, micturition interval and micturition duration values are expressed as mean±SEM. Statistical significance of differences between mean values of these parameters for control and autotransplanted rats was determined using the Student’s t-test. Statistical significance of differences between mean values of these parameters for control and 30 and 60 days for syngeneic transplanted rats was determined using Analysis of Variance (ANOVA) followed by the Bonferroni test to identify differences among the subgroups. A value of P<0.05 was considered statistically significant. Statistical analysis and graphs were done using the GraphPad Prism Program (GraphPad Prism 5.01, San Diego, CA, USA).

## RESULTS

### Post-operative follow-up

Out of ten autotransplanted rats two showed local infection and dehiscence of the abdominal wall suture three days after surgery and were excluded from the study. The eight remaining animals (80%) which survived the 30 day period exhibited a mean body increase of 35g without showing any complications. After the CMG determinations the omentum was removed from the bladder and no tissue discoloration, micro-abscesses, suture leakage or strictures were observed, and the sutures were entirely reabsorbed. Three animals had a single, friable calculus which did not adhere to the bladder wall or to the surgical site. The cystometrogram (CMG) data from these animals were not included in the quantitative analysis. Of 19 syngeneic transplanted rats two died within 24 hours after surgery; the remaining animals survived for 30 and 60 days uneventfully. Mean weight gain was 34g and 51g for the 30 day and 60 day transplanted rats, respectively. After the CMG determinations the omentum was removed from the bladder and similarly to the autotransplanted rats no tissue discoloration, micro-abscesses or strictures were observed. Intriguingly, no intravesical calculi were observed in these animals. In addition, no adherence to intestinal loops of the transplanted bladder or hydroureteronephrosis were observed in both groups of transplanted animals.

### Continuous Cystometrogram

**Figure 1:**
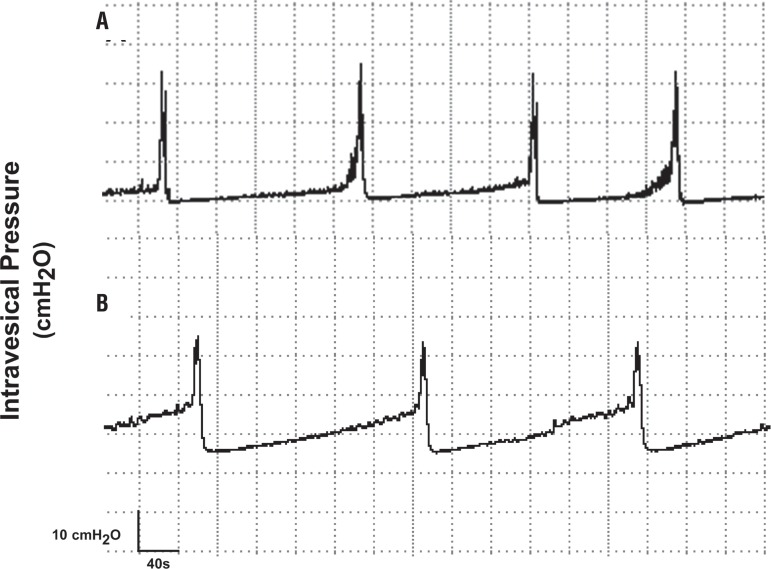
– Representative cystometric recordings of female Wistar rats which underwent bladder autotransplant. Non-operated rat (A) and rat 30 days after bladder autotransplant (B). The autotransplanted rat showed detrusor contractions, with amplitude of maximal pressure, micturition intervals and micturition duration similar to the normal rat. The threshold of the intravesical pressure was partially increased when compared with the recording of non-operated rat.

**Figure 2:**
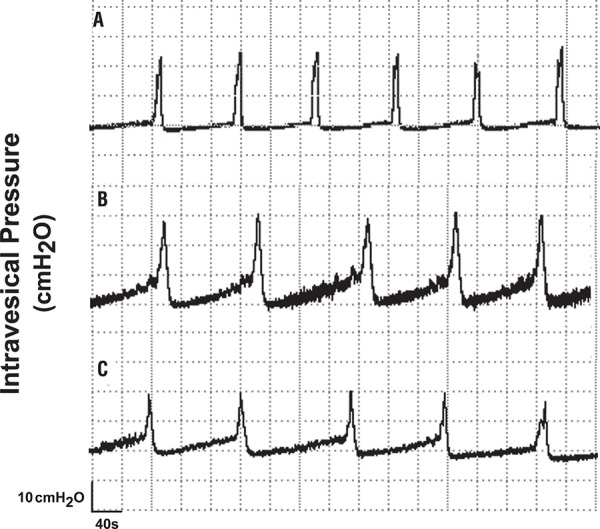
– Cystometric recordings of a female Wistar rat which underwent bladder autotransplant performed 30 days before. The rats were anesthetized with urethane for evaluation of the maximal pressure amplitude of the neobladder (A), micturition interval (B) and micturition duration (C). There was flow rate of bladder perfusion (0.9% saline solution, at 37 ºC) was 0.08 ml/min.

Figure-1 shows representative CMG tracings of an autotransplanted rat and of its respective control. Mean maximal intravesical pressure in autotransplanted rats was significantly lower than in control rats whereas mean duration of micturition and micturition intervals were not significantly different between control and autotransplanted rats (Figure-2 and Table-1). During the filling phase of the CMG, non-micturition contractions were not observed in either control or autotransplanted rats.

The asterisk indicates significant difference (P <0.05). Bl. = bladder; reimpl = autotransplant; d = days.

**Table 1:** Cystometric values in female Wistar rats that underwent urinary bladder autotransplant.

	Control (n=5)	Bladder Autotransplant (n=5)
Intravesical Pressure (cmH_2_O)	28.6 ± 2.7	23.0 ± 1.9
Micturition Interval (s)	238.1 ± 17.2	369.1 ± 25.5
Micturition Duration (s)	32.4 ± 1.3	34.1 ± 1.6

**Figure 3:**
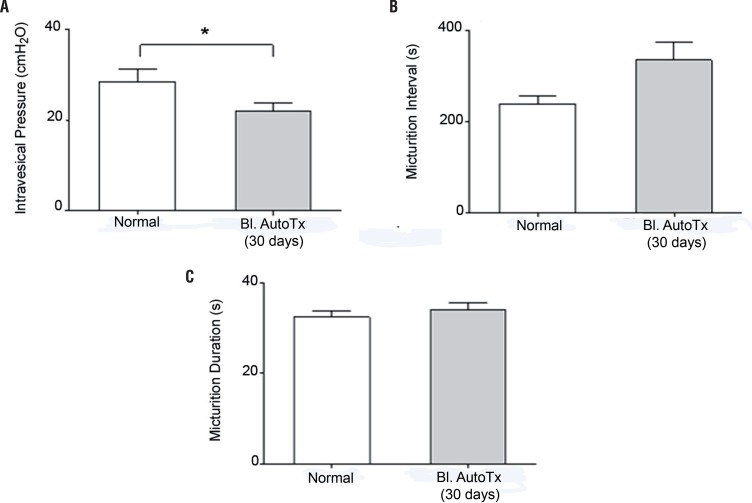
– Cystometric recordings of female Wistar rats which underwent bladder autotransplant performed 30 days after surgery. Evaluation of the maximal pressure amplitude of the bladder (A), micturition interval (B) and micturition duration (C). Flow rate of bladder perfusion (0.9% saline solution) was 0.08 ml/min (at 37ºC).

**Figure 4:**
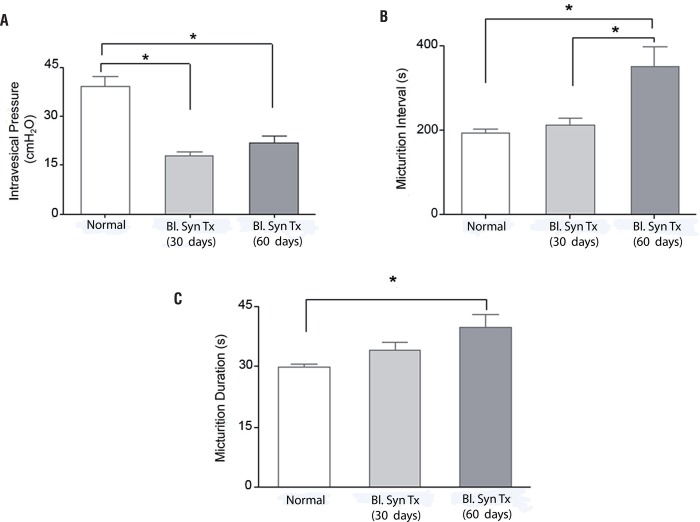
– Cystomanometric recordings of female SHR rats which underwent bladder transplantation performed 30 and 60 days after surgery. The rats were anesthetized with urethane for evaluation of the maximal pressure amplitude of the bladder (A), micturition interval (B) and micturition duration (C). Flow rate of the bladder perfusion (0.9% saline solution) was 0.08 ml/min (at 37ºC).

Figure-3 shows representative CMG tracings of control and syngeneic transplanted spontaneous hypertensive rats. Mean maximal intravesical pressure was significantly lower in the 30 and 60 day transplanted rats whereas mean micturition intervals were significantly different between normal rats and those 60 days after transplant and between rats 30 and 60 days after transplant, and mean micturition duration was significantly higher only in the 60 day transplanted animals (Figure-4 and Table-2). The data were also evaluated using Kolmogorov-Sminov test (KS) to test the Gaussian distribution. The results showed normal distribution.

*(P <0.05).

Bl. = Bladder; AutoTx. = Autotransplant

The asterisks indicate

significant difference (P <0.05).

Bl. = Bladder; Syn = Syngeneic; Tx = Transplant; d = days

**Table 2:** Cystometric values in female spontaneous hypertension rats (SHR) that underwent urinary bladder syngeneic transplant .

	Control (N=8)	Transplant (N=11) 30 Days	Transplant (N=6) 60 Days
Intravesical Pressure (cmH_2_0)	39.0 ± 3.1	18.3 ± 1.3	22.0 ± 1.9
Micturition Interval (s)	194.7 ± 8.5	214.0 ± 15.7	384.4 ± 25.8
Micturition Duration (s)	29.2 ± 0.9	34.1 ± 1.9	39.7 ± 3.3

**Figure 5:**
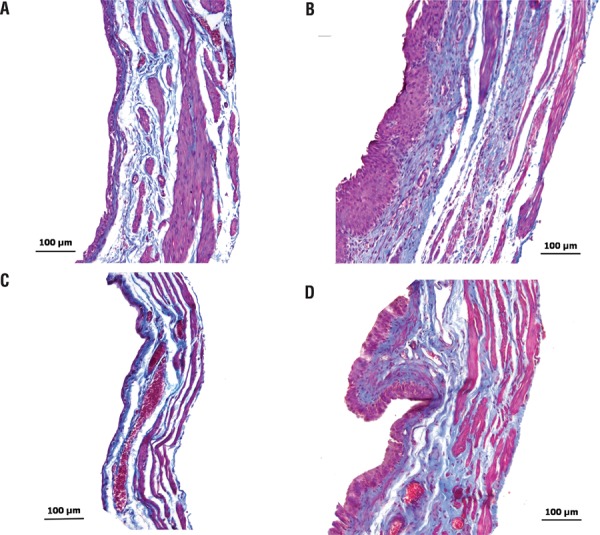
– Microphotography of sections of the bladder wall in female Wistar rats (A [control], B [autotransplanted bladder 30 days after surgery]), female SRH rats (C [control], and D [syngeneic transplanted bladder 30 days after surgery]). Different layers of the bladder wall show characteristics of preservation of the tissue, maintaining organization and architecture, without signs of fibrosis, wrinkles. There was increased thickening of the bladder wall, especially the epithelium which was thicker than that of the normal urinary bladder. Staining with Masson’s trichrome occurred as described in the experimental protocol.

The macroscopic configuration of neo-bladders was similar to the configuration of the normal bladder; the bladder wall was a little thicker but without signs of shrinkage, fibrosis or graft encrustation. Microscopically both autotransplanted and syngeneic transplanted bladders showed thickening of the epitelium, lamina propria and muscle layers (Figure-5).

## COMMENTS

The main finding of the present study consists in demonstrating that surgical procedures to transplant the supratrigonal bladder portion in rats are entirely feasible. This surgical procedure was associated with the preservation of not only the anatomical architecture of the bladder but also with its functional integrity. Although a similar procedure was previously reported by Yamataka et al. (19) these authors did not perform any urodynamic measurements to assess the functional status of the transplanted bladders. The fact that the maximal intravesical pressure developed during the micturition contraction was slightly lower in both groups of transplanted animals suggests that the postganglionic parasympathetic detrusor innervation was partially restored after the transplantation. Nevertheless, the low pressure generated by the detrusor contraction was sufficient to promote complete voiding of the bladder.

The fact that the transplanted bladders remained viable suggests that the reestablishment of their blood supply, most likely through angiogenesis, occurred rapidly. Wrapping the neobladder with the omentum may also have contributed to the rapid recovery of the irrigation of the transplanted bladders; the capacity of the omentum to provide angiogenic factors has been reported previously (14, 19-21). Indeed, it was observed that the omentum adherence to the bladder wall showed numerous blood vessels entering the grafted bladder. In preliminary experiments, it was also observed that the viability of bladder transplant was greatly impaired when no omental pouch was created. In addition, the fact that the excised supratrigonal bladder portion was kept in cold saline solution for only 30 min certainly contributed to the preservation and viability of the bladder tissue. Furthermore, total resorption of suture filaments and good cicatrization was observed in the suture site.

Since no surgical reconstruction of the blood vessels or nerve fibers of the autotransplanted or syngeneic transplanted bladder was performed, it is likely that endogenous angiogenic-(vascular endothelial growth factor-VEGF) and neurogenic-factors (nerve growth factor-NGF) were involved in the restoration of bladder irrigation and innervations (20-26) leading to the preservation of the micturition reflex.

## CONCLUSIONS

The major finding of the present study is that in the rat, supratrigonal bladder transplant is a feasible surgical procedure associated with full recovery of the micturition reflex. In addition, since preserved bladder function implies vascular and nerve regeneration of the transplanted organ this procedure could also be useful as an experimental model to investigate the mechanisms involved in angiogenesis as well as in peripheral nervous system neurogenesis.

## ARTICLE INFO

Int Braz J Urol. 2017; 43: 112-20
